# A Chikungunya Virus Multiepitope Recombinant Protein Expressed from the Binary System Insect Cell/Recombinant Baculovirus Is Useful for Laboratorial Diagnosis of Chikungunya

**DOI:** 10.3390/microorganisms10071451

**Published:** 2022-07-18

**Authors:** Leonardo Assis da Silva, Monique da Rocha Queiroz Lima, Brenda Rabello de Camargo, Dyeferson Kened da Silva Coelho Guimarães, Anabele Azevedo Lima Barbastefano, Raquel Curtinhas de Lima, Paulo Vieira Damasco, Rivaldo Venâncio da Cunha, Luiz José de Souza, Elzinandes Leal de Azeredo, Luzia Maria de-Oliveira-Pinto, Tatsuya Nagata, Daniel M. P. Ardisson-Araújo, Flavia Barreto dos Santos, Bergmann Morais Ribeiro

**Affiliations:** 1 Laboratory of Baculovirus, Cell Biology Department, University of Brasilia, Brasilia 70910-900, DF, Brazil; leocbq@gmail.com (L.A.d.S.); brendarc@gmail.com (B.R.d.C.); tatsuya@unb.br (T.N.); 2 Viral Immunology Laboratory, Oswaldo Cruz Institute Rio de Janeiro, Oswaldo Cruz Foundation, Rio de Janeiro 21040-900, RJ, Brazil; moniquerql@gmail.com (M.d.R.Q.L.); raquelcurtinhas@gmail.com (R.C.d.L.); elzinandes@ioc.fiocruz.br (E.L.d.A.); lpinto@ioc.fiocruz.br (L.M.d.-O.-P.); 3 Laboratory of Insect Viruses, Cell Biology Department, University of Brasilia, Brasilia 70910-900, DF, Brazil; ardissonaraujo@gmail.com; 4 Centro Universitário de Brasília, UniCEUB, Brasilia 70790-075, DF, Brazil; dyeferson1@gmail.com (D.K.d.S.C.G.); anabelebio@gmail.com (A.A.L.B.); 5 Pathological Anatomy, Gaffrée Guinle University Hospital Rio de Janeiro, Federal University of the State of Rio de Janeiro, Rio de Janeiro 20270-004, RJ, Brazil; paulovieiradamasco@gmail.com; 6 Rio-Laranjeiras Hospital, Rio de Janeiro 22240-000, RJ, Brazil; 7 Pedro Ernesto University Hospital, University of the State of Rio de Janeiro, Rio de Janeiro 20551-030, RJ, Brazil; 8 Medical Clinic Department, Federal University of Mato Grosso do Sul (UFMS), Campo Grande 79050-010, MS, Brazil; rivaldo_venancio@uol.com.br; 9 Plantadores de Cana Hospital, Campos dos Goytacazes, Rio de Janeiro 28025-496, RJ, Brazil; luizjosedes@gmail.com

**Keywords:** chikungunya, E2, recombinant protein, IgG-ELISA, baculovirus expression

## Abstract

Chikungunya virus (CHIKV) is an arbovirus currently distributed worldwide, causing a disease that shares clinical signs and symptoms with other illnesses, such as dengue and Zika and leading to a challenging clinical differential diagnosis. In Brazil, CHIKV emerged in 2014 with the simultaneous introduction of both Asian and East/Central/South African (ECSA) genotypes. Laboratorial diagnosis of CHIKV is mainly performed by molecular and serological assays, with the latter more widely used. Although many commercial kits are available, their costs are still high for many underdeveloped and developing countries where the virus circulates. Here we described the development and evaluation of a multi-epitope recombinant protein-based IgG-ELISA (MULTREC IgG-ELISA) test for the specific detection of anti-CHIKV antibodies in clinical samples, as an alternative approach for laboratorial diagnosis. The MULTREC IgG-ELISA showed 86.36% of sensitivity and 100% of specificity, and no cross-reactivity with other exanthematic diseases was observed. The recombinant protein was expressed from the binary system insect cell/baculovirus using the crystal-forming baculoviral protein polyhedrin as a carrier of the target recombinant protein to facilitate recovery. The crystals were at least 10 times smaller in size and had an amorphous shape when compared to the polyhedrin wild-type crystal. The assay uses a multi-epitope antigen, representing two replicates of 18 amino acid sequences from the E2 region and a sequence of 17 amino acids from the nsP3 region of CHIKV. The recombinant protein was highly expressed, easy to purify and has demonstrated its usefulness in confirming chikungunya exposure, indeed showing a good potential tool for epidemiological surveillance.

## 1. Introduction

After its recent global emergence, the chikungunya virus (CHIKV)—an arthritogenic arbovirus of family *Togaviridae* and genus *Alphavirus*—has spread across the five continents at an unprecedented rate and caused millions of cases, mostly in the tropical and subtropical regions of the world [1,2], with the most substantial burden on the Americas after 2010 [3]. The virus is a mosquito-borne virus discovered in Tanzania in 1952, which is vectored and spread by adult females of *Aedes aegypti* during a blood meal [4]. Four distinct genotypes are described, based on their geographical distribution, which include the West African, the East/Central/South African (ECSA), the Asian, and the Indian Ocean lineage (IOL) [5]. The CHIKV genome is a 11.8 kb positive-sense, single-stranded RNA consisting of two open-reading frames (ORFs) that encode four conserved nonstructural proteins (nsP 1–4), a capsid protein (C), two envelope glycoproteins (E1 and E2), and two other cleaved proteins (E3 and 6K). The nsPs of CHIKV are synthesized as a precursor polyprotein that is cleaved into nsP1, nsP2, nsP3, and nsP4. The nsP1, nsP2, and nsP4 are involved in RNA capping, helicase/protease activity, and polymerase activity, respectively [6]. On the other hand, the nsP3 plays a role in viral replication [7] and is involved in interactions with host proteins and signaling cascades [8], although its exact function remains unclear [9,10,11]. The E1 and E2 proteins are highly secreted in infected individuals during the acute phase of the disease [12,13], indeed, representing reliable markers for diagnostic approaches.

CHIKV infections can be asymptomatic or cause a discrete illness that ranges from a moderate to a severe condition. Fever, headache, fatigue, myalgia, rash, arthralgia, and arthritis are symptoms commonly observed during CHIKV infection [14]. However, in some patients, chronic polyarthritis may occur and remain for weeks or even years, after the acute phase [15]. Moreover, despite being rare, CHIKV infection has also been associated with neurological manifestations, such as Guillain–Barré’s syndrome and meningoencephalitis [16,17]. Anti-CHIKV IgM antibodies are elicited as early as three days after viral infection, but its presence is usually only detected three to four months later [18]. Anti-CHIKV IgG antibodies, in contrast, remain detectable in convalescent individuals for many years [19].

In Brazil, CHIKV autochthonous transmission occurred after the simultaneous introduction of both the Asian and the ECSA genotypes in 2014 [20], with the latter more frequently associated with symptomatic cases [21,22,23]. Since then, the virus has been co-circulating with other arboviruses, such as dengue virus (DENV), Zika virus (ZIKV), yellow fever virus (YFV), and Mayaro virus (MAYV), that when combined have increased the incidence of exanthematic-related diseases [24]. In areas where those arboviruses co-circulate, suspected cases can be misdiagnosed and remain significantly underreported due to the similar overlap of the clinical signs and symptoms [25]. The arboviruses co-circulation was reported to lead to an underestimation in the Zika cases in the Americas, which were more common in countries, such as Brazil, that present with a higher incidence of dengue and chikungunya infection cases [26]. In that scenario, an accurate laboratorial differential diagnosis is crucial to evaluate the impact of those infections, especially during these overlapping periods [27].

Commercial kits for the serological diagnosis of chikungunya are available; however, their costs represent a financial burden for many of the chikungunya endemic countries. In-house IgG-ELISA has been reported as an accurate and reliable approach to characterize the human immune responses in CHIKV infections [1]. Moreover, the detection of specific anti-CHIKV IgG antibodies is a consistent strategy used in seroprevalence studies in those areas where DENV and ZIKV co-circulate [19,28,29,30]. However, in-house ELISAs, a more affordable approach, require viral antigens produced in a limited quantity, by the growth of CHIKV in the brains of mice, which is insufficient for the large-scale serological screening necessary for the spread of the virus. In addition, the use of whole viruses or crude extracts poses a potential health risk through exposure to infectious viral particles and may cause a cross-reaction with the antibodies against the Mayaro virus which circulates in Brazil. To overcome this issue, we developed and evaluated an in-house ELISA for the detection of anti-CHIKV IgG antibodies using a recombinant protein produced from baculovirus-infected insect cells as the antigen. The protein forms an easily-purified crystal that carries a multi-epitope polypeptide from CHIKV. Several recombinant protein expression systems have been used in arboviruses multi-epitope antigen preparations [31,32,33], including the baculovirus expression vector system in insect cells that yields high levels of proteins [33,34,35]. Here we sought to investigate the efficiency of a CHIKV multi-epitope protein easily produced and purified, for the serodiagnosis of chikungunya and seroprevalence studies.

## 2. Materials and Methods

### 2.1. Construction of Recombinant Baculovirus Containing R1 Gene

**Figure 1:**
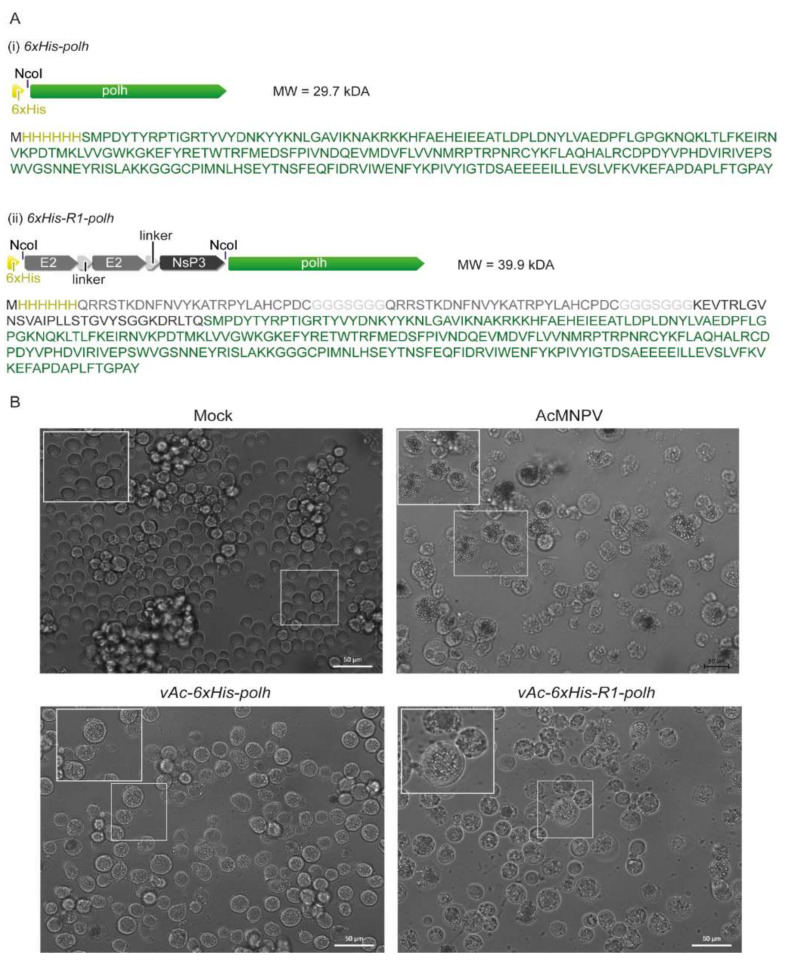
Schematic representation and light microscope of recombinants 6xHis-polh and 6xHis-R1-polh. (**A**) Schematic representation of modified polh gene cloned into pFastBac1 to generate 6xHis-polh and CHIKV multi-epitope gene (R1) fused at 5′ region of polh (6xHis-R1-polh); (**B**) Sf9 cells at 72 h.p.i. with the respective second passage virus stock (m.o.i. of five): mock-infected cells, cells infected with vAc-6xHis-polh, vAc-polh-R1-6xHis, and AcMNPV. The insets show details of infected cells with the amorphous crystal that accumulate in nucleus of the infected cells.

The CHIKV multi-epitope gene (R1) that contains an 18 amino acid sequence from the E2 region and a sequence of 17 amino acids from the nsP3 region (Figure 1A) was synthetized by the company, Integrated DNA Technologies (IDT). The R1 gene was cloned into the *Nco*I restriction enzyme site of the vector pFastBac1-6xhis-polh [36,37]. After that, the donor pFastBac1-6xhis-R1-polh was transformed in *E. coli* DH10Bac cells (Thermo Fisher Scientific, Waltham, MA, USA) by electroporation and the R1 gene, under control of the polh promoter, was inserted into the genome of Autographa californica multiple nucleopolyhedrovirus (AcMNPV) via transposition (Bac-to-Bac^®^, Baculovirus Expression Systems, Thermo Fisher Scientific, Waltham, MA, USA), generating the recombinant bacmid vAc-R1-6xHis. All of the procedures followed the manufacturers’ recommendations. To produce infectious baculoviruses, one microgram of vAc-R1-6xHis DNA was transfected into lepidopteran (Spodoptera frugiperda) Sf9 cells (10^6^) grown in a six-well plate using FuGENE^®^ HD Transfection Reagent (Promega, Madison, WI, USA) and following the manufacturer’s protocol. The Sf9 cells were kept at 27 °C in TC-100 medium (Vitrocell, Campinas, São Paulo, Brazil) supplemented with 10% fetal bovine serum, 25 µg/mL amphotericin B, and 50 mg/L gentamycin sulfate. 

Seven days post-transfection, the supernatant was collected and the recombinant virus was titrated, as described elsewhere [38]. Then, Sf9 cells (1.5 × 10^7^) grown in 75 cm^2^ flasks were infected at a multiplicity of infection (M.O.I.) of one to increase the virus titer, as described in O’Reilly et. al. [38]. The infected Sf9 cells presented typical cytopathic effects, such as increasing nucleus size and presence of OBs (Figure 1B).

### 2.2. Expression and Recombinant OBs Purification

The suspension Sf9 cells were cultured in 100 mL supplemented TC-100 medium (in 250-mL glass flasks) at densities varying between 1.5–2 × 10^6^ cells/mL and then infected at M.O.I of 10 with two recombinant viruses (vAc-6xHis-polh and vAc-polh-R1-6xHis) and one wild-type virus (AcMNPV). After 72 h post-infection (h.p.i.), the cells were harvested by centrifugation at 7000× *g* for 10 min. The pellet from each flask was resuspended in the same volume of 5% Triton X-100 and centrifuged at 7000× *g* for 10 min; these procedures were repeated twice. The resulting pellet was resuspended in 0.5 M NaCl, centrifuged as above, and resuspended with Phosphate Buffered Saline (PBS) (137.0 mM NaCl, 2.7 mM KCl, 10.0 mM Na_2_HPO_4_, 2.0 mM KH2PO4, pH 7.4), supplemented with 1 mM phenylmethylsulfonyl fluoride (PMSF) protease inhibitor, and sonicated twice for 30 s at 25% amplitude. The suspended solution was loaded into a discontinuous sucrose gradient (40–80% of sucrose in PBS) and centrifuged at 130,000× *g* for 2 h. The band containing the putative recombinant OBs were removed from the gradient, five-fold diluted with PBS, and centrifuged at 7000× *g* for 10 min. The purified recombinant OBs were subjected to SDS-PAGE and ultrastructural analysis.

### 2.3. Scanning Electron Microscopy and Recombinant OBs Synthesis Analysis

The recombinant OBs (AcMNPV, vAc-6xHis-polh, and vAc-6xHis-R1-polh), were mixed with the same volume of 100% acetone and placed in a metallic support “stub” covered with double-sided carbon tape and kept in an oven at 37 °C until completely dried. The stubs were coated with gold in a Sputter Coater (Oerlikon Balzers, Balzers, Liechtenstein), following the manufacturer’s instructions, and then observed in a SEM Jeol JSM 840A (JEOL, Tokyo, Japan) at 10 kV.

For the SDS-PAGE, 45 µL of suspension of each recombinant OBs were added to a volume of 15 µL per sample in four × protein loading buffer (0.25 M Tris-Cl, pH 6.8, 4% SDS, 20% glycerol, 10% 2-mercaptoethanol, and 0.02% bromophenol blue), heated (100 °C) for 5 min, and run through a 12% polyacrylamide gel using the Mini-Protean Tetra Cell (Bio-Rad, Hercules, CA, USA). The proteins were visualized by Coomassie brilliant Blue R-250 staining. For the Western blotting, the proteins were transferred onto Immobilon-P transfer membrane (MilliporeSigma, Burlington, MA, USA) using a Trans-Blot Semi-Dry Transfer Cell (Bio-Rad, Hercules, CA, USA). The membranes were then blocked in one × PBS Buffer (137 mM NaCl, 2.7 mM KCl, 10 mM Na2HPO4, 2 mM KH2PO4, pH 7.4) containing 3% bovine serum albumin for 16 h at 4 °C. Then, they were washed three times with one × PBS Tween (0.05%). The primary and secondary antibodies were incubated for one h and washed three times with one × PBS Tween (0.05%) between incubations. Chromogenic substrate NBT-BCIP (Promega) was used for the detection of N protein, according to the manufacturer’s protocol.

### 2.4. Ethical Statement and Human Serum Samples

The samples analyzed in this study were from an ongoing project approved by the Oswaldo Cruz Foundation Ethics Committee (CAAE 57221416.0.1001.5248). The patients’ personal information was anonymized before the data were accessed. The serum samples used in this study belong to a previously gathered collection of the Viral Immunology Laboratory at the Oswaldo Cruz Institute, Rio de Janeiro, Brazil, from epidemics that occurred from 1997 to 2019. The CHIKV-positive infection samples were identified through clinical symptoms (fever), confirmed by CHIKV RT-qPCR [39], and the detection of IgM and/or IgG anti-CHIKV antibodies by capture ELISAs (Euroimmun Anti-Chikungunya virus IgM and IgG kits, catalogue numbers EI 293a M and EI 293a G, respectively; Euroimmun, Lubeck, Germany). Individuals were classified as negative for CHIKV infection if the results were negative by all of the methods described above, as recommended when testing for chikungunya according to the WHO/PAHO criteria [40]. A panel of 495 serum samples was divided into eighteen groups: Groups A and B, sera serologically confirmed for CHIKV specific IgM (*n* = 77) and IgG (*n* = 22); Group C, CHIKV positive cases by RT-qPCR (*n* = 62); Groups D–G, sera confirmed for DENV-1 (*n* = 30); DENV-2 (*n* = 30); DENV-3 (*n* = 30) and DENV-4 (*n* = 30); Group H, IgM positive and IgG negative dengue cases (*n* = 30); Group I, dengue IgG positive cases (*n* = 04); Group J, Zika positive cases (*n* = 35); Group K, sera yellow fever positive (*n* = 10); Group L, sera from individuals vaccinated for yellow fever (*n* = 24); Group M, sera from measles patients (*n* = 12); Group N, sera from rubella patients (*n* = 12); Group O, sera from hepatitis C patients (*n* = 10); Group P, sera from leptospirosis patients (*n* = 12); Group Q, sera from healthy individuals (*n* = 22); Group R, sera of symptomatic suspected cases for arboviral infection (dengue), but considered as negative after routine laboratorial diagnosis, *n* = 43). Briefly, the routine differential diagnosis was based on MAC-ELISA (dengue IgM Capture ELISA, E-DEN01M, Panbio, Brisbane, Australia); IgG-ELISA, according to Miagostovich et al. [41]; NS1-ELISA (PlateliaTM Dengue NS1 Ag-ELISA kit, Biorad Laboratories, Marnes-La-Coquette, France); and/or RT-PCR according to Lanciotti et al. [42] for dengue diagnosis. The Zika investigation was performed by using RT-qPCR, according to Lanciotti et al. [43]. For the sensitivity and specificity analyses, the Euroimmun Anti-Chikungunya virus IgG kit (Euroimmun, Lübeck, Germany) was used, and Group B was established as a positive control for CHIKV, which represents reactive patient sera for the specific IgG anti-CHIKV. Group Q was considered negative as it did not react to the specific IgG anti-CHIKV. Therefore, “true positive” Group B and “true negative” Group Q were established.

### 2.5. Multiepitope Recombinant Protein-Based IgG-ELISA for the Detection of Anti-CHIKV Specific IgG Antibodies (MULTREC IgG-ELISA)

The MULTREC IgG-ELISA was performed, as previously described by Dos Santos et al. [44], with modifications. Microplates of 96-wells (Immulon Dynatech Industries, Inc., Chantilly, VA, USA) were coated with 100 µL of a mixture of recombinant CHIKV multi-epitope gene (R1) (6 µg/mL each per well) and incubated overnight at 4 °C. The plates were blocked with 1× PBS pH 7.4, 5% nonfat drink milk, 3% goat serum, 3% fetal bovine serum (FBS), 1% BSA, and 20% Tween 20 for 1 h at 37 °C. One hundred microliters of 1:40 diluted patients’ serum was added with the same blocking solution; the plates were incubated at 37 °C for 2 h. Posteriorly, 40 µL of conjugated anti-human IgG with horseradish peroxidase (Sigma-Aldrich) diluted 1:5000 was added. Between all of the incubation steps, the plate was washed in 1× PBS pH 7.4. After 30 min at 37 °C, 100 µL/well of 2,2-azino-di-3-ethyl-benzothiazoline sulfonate substrate (Kirkegaard and Perry Laboratories, Gaithersburg, MD, USA) was added. The plates were incubated for 30 min at room temperature and the optical density (OD) was measured at 405 nm. Each serum sample was tested in duplicate wells, uncoated or coated with R1, and negative and positive controls were included in each plate. The cutoff OD value for seropositivity was set at ≥0.3503 since this value was consistently above the average adjusted OD plus three standard deviations for negative control sera.

## 3. Results

### 3.1. Fusion of the CHIKV Multi-Epitope Gene-Coding Sequence at Amino-Terminal Region of the Modified Polh

We fused the CHIKV multi-epitope gene (R1) at the amino-terminal region of the modified polh to generate OBs containing R1 epitopes in recombinant virus-infected insect cells. As a proof-of-concept, this is an attempt to facilitate antigen purification to be further used in CHIKV diagnosis. To do so, we generated a recombinant baculovirus, vAc-6xHis-R1-polh, that contained the CHIKV multi-epitope gene-coding sequence at the 5′-end of the modified *polh* version (Figure 1A). The recombinant virus was used to infect Sf9 cells in vitro, which developed cytopathic effects, such as nucleus hypertrophy and the production of crystals (Figure 1B).

### 3.2. Ultrastructural Analysis of the CHIKV Antigen-Containing Recombinant Crystal Revealed Smaller Crystals Than the Parental Version

**Figure 2:**
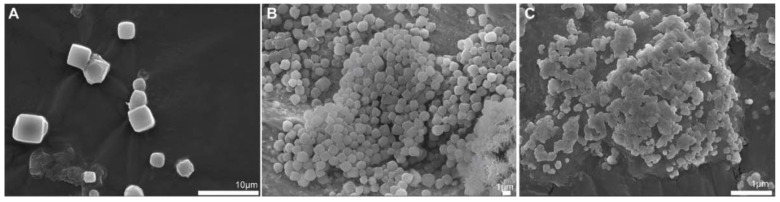
Scanning electron microscopy (SEM) of the purified recombinant OBs. (**A**) Native AcMNPV OB; (**B**) 6xHis-polh OBs; and (**C**) 6xHis-R1-polh OBs (Scale bar = 1 or 10 µm).

We found that the R1 multi-epitope fused to the crystal-forming protein polyhedrin affected both the shape and the size of the recombinant crystals (Figure 2). To do so, we subjected the recombinant crystals produced from the infected Sf9 cell to scanning electron microscopy. The R1 fusion version produced discrete recombinant crystals with polyhedral-like shapes that resembled the wild-type AcMNPV OBs (Figure 2A) and the parental 6xhis-polh version (Figure 2C). The R1 crystals presented amorphous shapes and sticky surfaces when compared to the parental crystal version, with a size at least 10-fold smaller (Figure 2). 

### 3.3. CHIKV Antigen-Containing Recombinant Crystal Immunoblot

**Figure 3:**
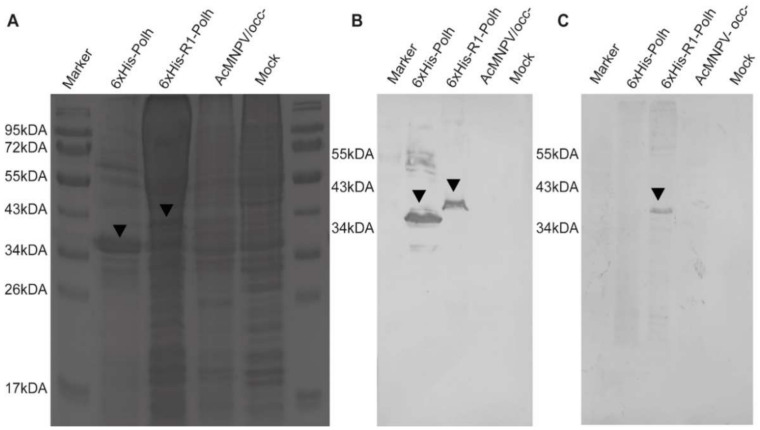
Protein expression analyses of the recombinant 6xHis-R1-polh in insect cells infected with different recombinant baculoviruses, including vAc-6xHis-polh, vAc-6xHis-R1-polh, and AcMNPV/occ- (without polh expression). (**A**) SDS-PAGE of insect cells extracts infected with different recombinant baculoviruses; (**B**,**C**) Membranes immune-stained with (**B**) anti-6xHis (Promega), (**C**) anti-CHIKV-positive patient’s serum as primary antibodies, incubated with mouse and human anti-IgG, respectively, and conjugated to alkaline phosphatase enzyme (Invitrogen). The proteins’ reacting bands were detected using the substrate NBT/BCIP (Promega). Black arrowheads point the specified bands.

We carried out an immunoblot assay to confirm the construction of the recombinant crystals containing the R1 multi-epitope. We found that the R1 recombinant crystals could be recognized by both an anti-his and a human CHIKV-positive serum. As expected, the anti-his antibody recognized the two crystals 6xHis-R1-polh and 6xHis-polh, but failed to recognize the wild-type AcMNPV OB. The reactive bands corresponded to the theoretical weight mass of the fusion version vAc-6xHis-R1-polh (about 40 kDa), and also the parental version vAc-6xHis-polh (about 30 kDa). On the other hand, a human CHIKV-positive serum was able to recognize only the R1-containg version of the chimerical protein, which confirmed the lack of cross reaction with the polh (Figure 3).

### 3.4. Multiepitope Recombinant Protein-Based IgG-ELISA for the Detection of Anti-CHIKV Specific IgG Antibodies (MULTREC IgG-ELISA)

**Figure 4:**
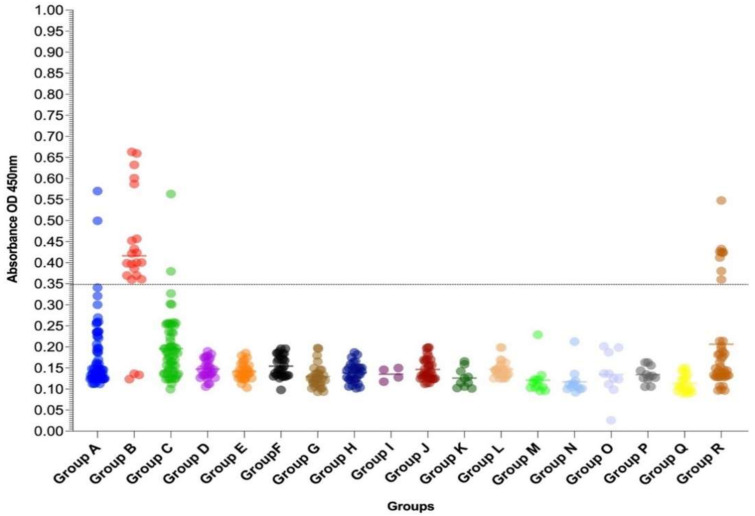
IgG antibody response in chikungunya patients and control groups (*n* = 495) to the chikungunya recombinant polypeptide as determined by the MULTREC IgG-ELISA. Absorbance values obtained using the MULTREC IgG-ELISA on a panel (*n* = 495) of chikungunya cases and controls. (-) represents the mean value for each group and (---) dashed lines, the cut-off value for the test (0.3503) as determined at a wavelength of 450 nm and a reference value between 620 nm and 650 nm.

We further investigated the usefulness of the R1 recombinant crystals as the antigen source for the laboratorial diagnosis of chikungunya, using a panel of well-characterized samples, in an IgG-ELISA (MULTREC IgG-ELISA). The chikungunya IgG positive cases, analyzed in Group B (days 1 to 32 after the onset of symptoms), were previously tested by the Euroimmun Anti-Chikungunya virus IgG kit during the 2018–2019 epidemic in Rio de Janeiro, Brazil, and, therefore, considered as true positive (Table 1). In these chikungunya IgG positive cases (Group B), the MULTREC IgG-ELISA was very sensitive and confirmed 86.36% (19/22) of the tested samples (Table 1 and Figure 4).

In the chikungunya IgM positive cases (Group A), the MULTREC IgG-ELISA confirmed 3.89% (3/77) of the cases, while all of them were negative when evaluated using the commercial kit. As expected, a low sensitivity was also observed in the acute CHIKV cases, positive by the RT-qPCR (Group C; 3.22%; 2/62) (Table 1 and Figure 4).

Based on the analysis of the sera from healthy individuals (Group Q), considered true negative, the specificity for the Anti-Chikungunya virus IgM ELISA kit was 100% (Table 1). However, on the sera of the symptomatic suspected cases for arboviral infection (dengue), considered as negative after differential diagnosis (Group R), the test was positive in 18.60% (8/43) of the cases, but most of those were close to the MULTREC IgG-ELISA cut-off value (Figure 4). No cross-reactivity was observed with sera from the dengue IgG positive patients (Group I), positive for Zika (Group J), yellow fever (Group K) or those vaccinated against yellow fever (Group L), positive for rubella (Group M), measles (Group N), hepatitis C (Group O) and leptospirosis (Group P) (Table 1 and Figure 4).

## 4. Discussion

The co-circulation of arborviruses leads to a challenging clinical diagnosis, due to the signs and symptoms shared by those viruses. Therefore, a reliable laboratorial diagnosis is critical for the disease surveillance and clinical management [45,46]. It has been shown that there are still gaps in arboviruses seroprevalence studies, and the importance in developing sensitive and specific diagnostic tools for those studies has been stressed [29]. In this sense, serological techniques are widely available, easier to perform, and relatively cheaper than molecular approaches [47].

Currently, several commercial kits for the serological diagnosis of chikungunya are available, with some of them with good sensitivity and specificity [48,49,50,51]. However, the high cost of production and the low specificities due to the presence of other arboviruses are still a concern for many countries where the disease occurs. A recent study, for example, evaluated a commercial IgM ELISA test, routinely used for chikungunya diagnosis in Brazil, a dengue-endemic area, and, despite the high sensitivity, the test presented low specificities, due to the cross reactivities observed with dengue [52].

Although the IgM antibody is the marker of choice in the diagnosis of chikungunya, some of the patients with CHIKV infections produce low or undetectable levels of IgM, and the IgM response may be slow to appear and be short-lived [18,49]. Previous studies have shown that the IgG is a more sensitive marker than IgM, in CHIKV infections after a DENV infection [53,54], and the MULTREC IgG-ELISA has proven to be highly reliable for the diagnosis of those cases. Moreover, it is the marker of choice for seroprevalence studies [19,28,29,30].

The recombinant multi-epitope antigen produced in this work consists of two replicates of 18 amino acid sequences from the E2 region and a sequence of 17 amino acids from the nsP3 region of CHIKV, that are highly expressed, easy to purify, and have demonstrated high sensitivity and specificity in the diagnosis of chikungunya. The E1 and E2 proteins from CHIKV are those most frequently used by serological diagnostic kits [55], and the E2 is considered to be more immunodominant and, thus, with a higher diagnostic potential in comparison to E1 [56,57]. In fact, it has been shown that a recombinant CHIKV E2-based ELISA was more sensitive and specific than a CHIKV E1-based one [58]. In E2 and E3, the capsid and nsP3 proteins are the targets of the anti-CHIKV antibody response [59].

Several studies have shown the usefulness of the CHIKV E2 for the serological diagnosis of chikungunya, using prokaryotic and eukaryotic expression systems [55,60,61,62]. Recently, a study using recombinant CHIKV E2 antigens, produced in both prokaryotic and eukaryotic expression systems, showed a strong reaction to anti-CHIKV IgG antibodies, with an accuracy higher than 90% [62]. An assay for chikungunya diagnosis, using recombinant multi-epitope proteins expressed in plants, reported a good quality expression and confirmation by Western-blotting [63]. The same multi-epitope strategy, using an eukaryotic system, was used to produce recombinant antigens, useful for the differentiation between dengue and Zika infections [33].

Recombinant chikungunya antigens produced in baculovirus/insect cell-based expression systems effectively address the issues related to biological risks, costs, and specificity associated with the use of diagnostic tests based on virus antigens produced in murine models and culture systems, that are laborious and time-consuming. Moreover, as immunodominant and specific protein regions may be selected using those expression systems, the cross-reactivities in the assays may be reduced. The IgG ELISAs, such as the one established here, use lower amounts of recombinant antigens when compared to the IgM capture ELISAs, for instance, increasing its usefulness for large scale testing. Based on the results and the studies discussed here, the recombinant protein-based ELISAs are shown to be a safe and cost-effective method for the diagnosis of chikungunya and seroprevalence studies. Moreover, IgG ELISAs, such as the one established here, use lower amounts of recombinant antigens when compared to the IgM capture ELISAs, for instance.

One limitation of this study is that we did not address the cross-reactivity against Mayaro (MAYV), Venezuelan equine encephalitis (VEEV), and the Eastern equine encephalitis (EEEV) viruses, but this shall be further evaluated, in particular for use in the areas where those viruses circulate. However, even though MAYV and CHIKV belong to the same genus and family, a previous study showed no cross-reaction when a recombinant CHIKV E2 expressed in the *Escherichia coli* system was tested against MAYV [61]. Nevertheless, the development of differential diagnoses for arboviruses is essential, and relies on specific and accurate diagnostic methods.

## Figures and Tables

**Table 1:** Evaluation of the MULTREC IgG-ELISA for chikungunya diagnosis based on the analysis of the distinct Groups.

GROUPS ^a^	Year	REC IgG-ELISA Result/Tested (%)		Kit Euroimmun Anti-Chikungunya Virus IgG Result/Tested (%)	
		NEGATIVE	POSITIVE	NEGATIVE	POSITIVE
A (CHIKV IgM positive cases, *n* = 77)	2018–2019	74/77 (96.10)	3/77 (3.89)	77/77 (100)	0/77 (0)
B (CHIKV IgG positive cases, *n* = 22)	2018–2019	3/22 (13.63)	19/22 (86.36)	0/22 (0)	22/22 (100)
C (CHIKV qRT-PCR positive cases, *n* = 62)	2018–2019	60/62 (96.77)	2/62 (3.22)	59/62 (95.16)	3/62 (4.83)
Total for Groups A–C, *n* = 161		137/161 (85.09)	24/161 (14.90)	136/161 (84.47)	25/161 (15.52)
D (DENV-1 cases, *n* = 30)	1997–2001	30/30 (100)	0/30	30/30 (100)	0/30
E (DENV-2 cases, *n* = 30)	1998–2010	30/30 (100)	0/30	30/30 (100)	0/30
F (DENV-3 cases, *n* = 30)	2001–2007	30/30 (100)	0/30	30/30 (100)	0/30
G (DENV-4 cases, *n* = 30)	2012–2017	30/30 (100)	0/30	30/30 (100)	0/30
H (DENV IgM positive and IgG negative cases, *n* = 30)	1997–2004	30/30 (100)	0/30	30/30 (100)	0/30
I (DENV IgG positive cases, *n* = 04)	1999–2001	4/4 (100)	0/4	4/4 (100)	0/4
J (ZIKV qRT-PCR positive cases, *n* = 35)	2016–2017	35/35 (100)	0/35	35/35 (100)	0/35
K (YFV cases, *n* = 10)	1997–2004	10/10 (100)	0/10	10/10 (100)	0/10
L (YFV vaccinee cases, *n* = 24)	1999–2019	24/24 (100)	0/24	24/24 (100)	0/24
M (Rubella cases, *n* = 12)	2004	12/12 (100)	0/12	12/12 (100)	0/12
N (Measles cases; *n* = 12)	2004	12/12 (100)	0/12	12/12 (100)	0/12
O (Hepatitis C cases, *n* = 10)	2013	10/10(100)	0/10	10/10 (100)	0/10
P (Leptospirosis cases, *n* = 12)	Not available	12/12 (100)	0/12	12/12 (100)	0/12
Q (Healthy individuals, *n* = 22)	Not available	22/22 (100)	0/22	22/22 (100)	0/22
R (Arboviruses negative cases, *n* = 43)	2000–2004	35/43 (81.39)	08/43 (18.60)	38/43 (88.37)	05/43 (11.62)
Total for Groups D–R, *n* = 334		326/334 (97.60)	08/334 (02.39)	329/334 (98.5)	05/334 (1.49)

^a^ Individuals in Groups A to C had confirmed CHIKV infection; Individuals in Groups D to R had no CHIKV infection.

## Data Availability

All data generated during this study are included in this published article.
